# Supplementary material for: Towards Standardisation of a Diffuse Midline Glioma Patient-Derived Xenograft Mouse Model Based on Suspension Matrices for Preclinical Research
Source: Biomedicines. 2023 Feb 11;11(2):527. doi: 10.3390/biomedicines11020527 (PMC9952880; doi:10.3390/biomedicines11020527)
Supplement: Supplementary file 1 [file biomedicines-11-00527-s001.zip › Supplementary Figures.pdf]

Supplementary Figures

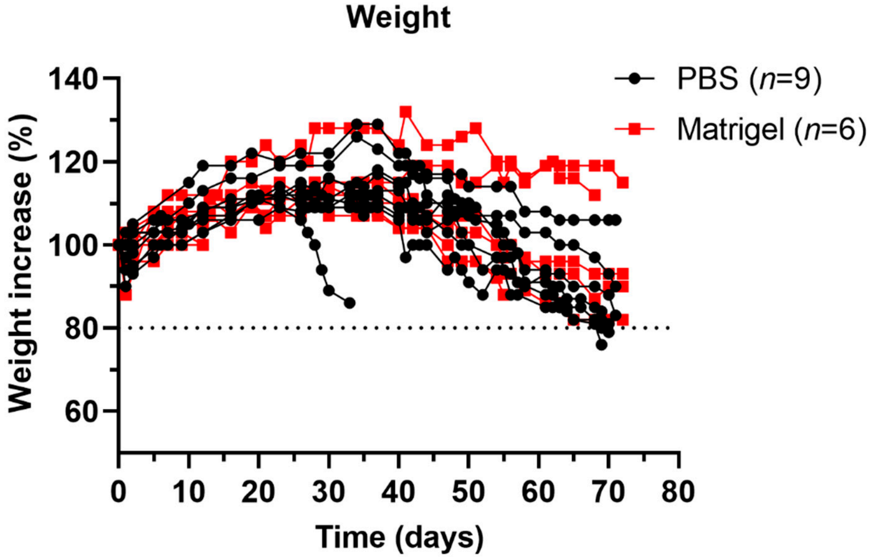

Supplementary Figure S1: Weight profiles of individual mice after inoculation with HSJD-DIPG-007 cells suspended in PBS or Matrigel. Weight was monitored until terminal endpoint. Dotted line represents humane endpoint threshold (representing 20% weight loss).

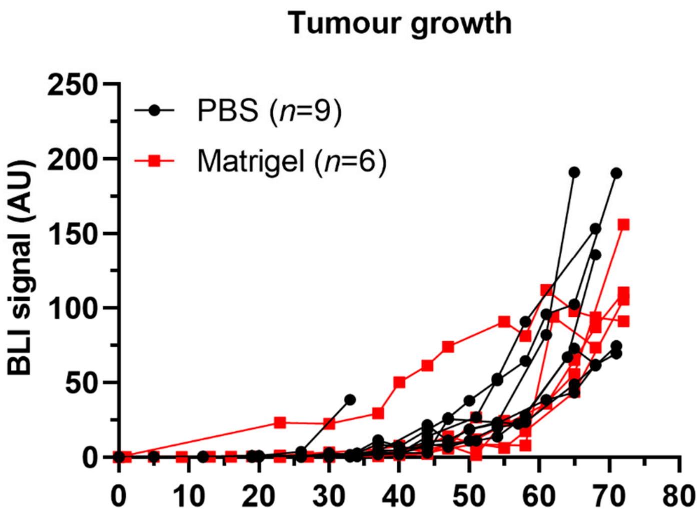

Supplementary Figure S2: Tumour growth profiles of individual mice following inoculation with HSJD-DIPG-007 cells suspended in PBS or Matrigel. Individual tumour volume over time in both PBS and Matrigel suspension groups shows comparable tumour growth up to 75 days.

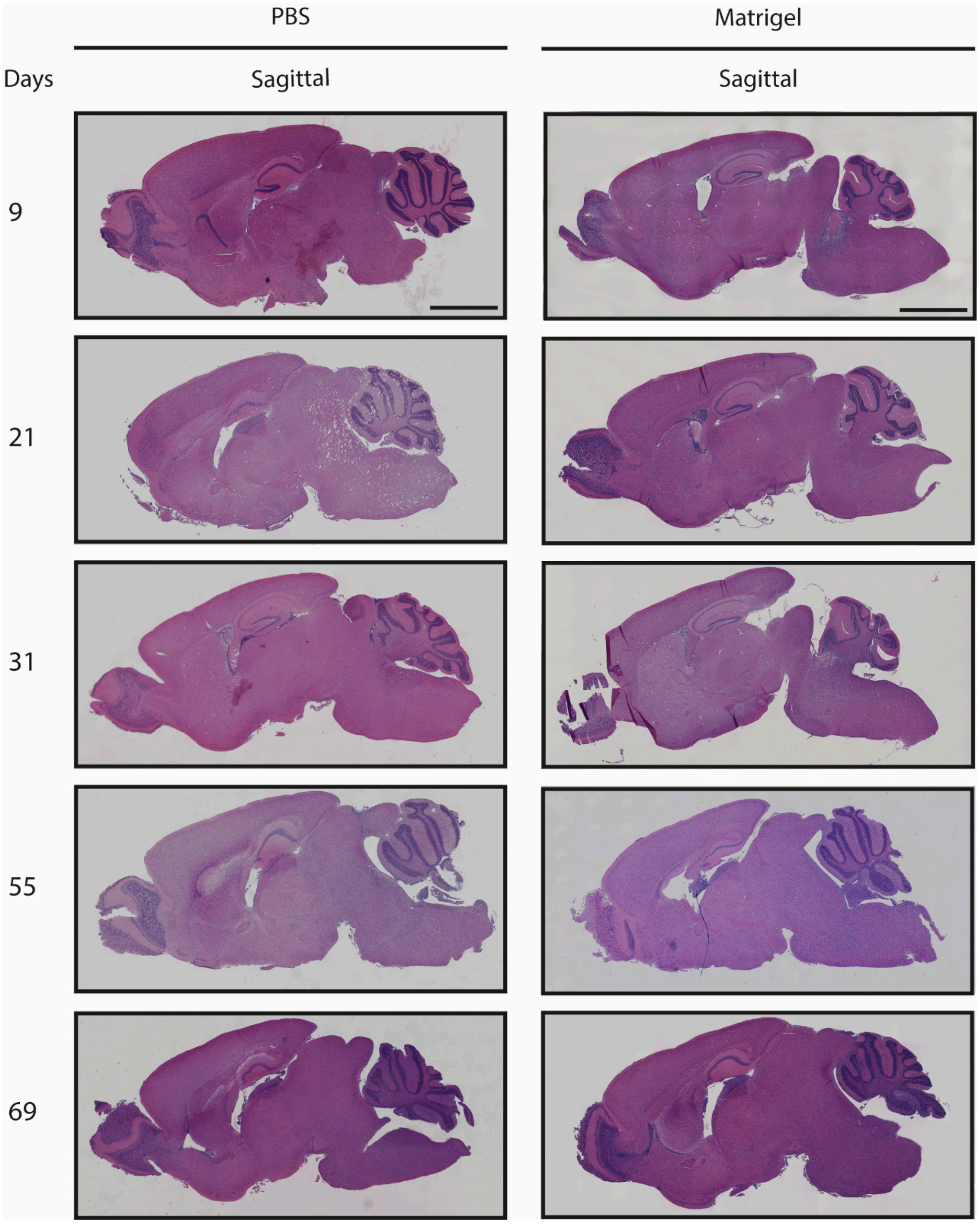

Supplementary Figure S3: Haematoxylin & Eosin staining of mouse brains with tumour progression over time following inoculation with HSJD-DIPG-007 cells suspended in PBS or Matrigel. No apparent histological changes were observed over time. Scale bar = 2mm.
