# Supplementary material for: Towards Standardisation of a Diffuse Midline Glioma Patient-Derived Xenograft Mouse Model Based on Suspension Matrices for Preclinical Research
Source: Biomedicines. 2023 Feb 11;11(2):527. doi: 10.3390/biomedicines11020527 (PMC9952880; doi:10.3390/biomedicines11020527)
Supplement: Supplementary file 1 [file biomedicines-11-00527-s001.zip › Supplementary Table.pdf]

Supplementary Table S1: List of conditions, materials, and manufacturers related to the in vivo experiments including living conditions, cage enrichments, food, water, and health monitoring of the animals by pathogen detection in the research facility.

**Parameter**	**Condition/Material**	**Manufacturer**
Temperature	21 ± 1°C	-
Relative Humidity	45 – 64%, TouchSLIM Plus™ AHU	Tecniplast S.p.A., Buguggiate, Italy
Lighting	12h light/dark (7 a.m. – 7 p.m.)	-
Housing	Specific Pathogen Free	-
Caging	GM500 Mouse IVC (Green Line)	Tecniplast S.p.A., Buguggiate, Italy
Mice/cage	Up to 4	-
Bedding	Aspen Wooden flakes	Bio Services BV, Uden, The Netherlands
Diet	ssniff® R/M-H (V153x), *ad libitum*	Ssniff Spezialdiäten GmbH, Soest, The Netherlands
Water	Sterile, *ad libitum*	Aqua B. Braun, Melsungen, Germany
Enrichment	Tissue	WEPA Professional GmbH, Müschede, Germany
	Mouse Tunnel	Bio-Serv, San Diego USA
Pathogen detection	Dirty-bedding sentinels	-
